# A systematic review investigating fatigue, psychological and cognitive impairment following TIA and minor stroke: protocol paper

**DOI:** 10.1186/2046-4053-2-72

**Published:** 2013-09-08

**Authors:** Grace M Moran, Benjamin Fletcher, Melanie Calvert, Max G Feltham, Catherine Sackley, Tom Marshall

**Affiliations:** 1 Primary Care Clinical Sciences, University of Birmingham, Edgbaston, Birmingham, B15 2TT, England; 2 Faculty of Medicine and Health, University of East Anglia, Norwich Research Park, Norwich, NR4 7TJ, England

**Keywords:** Transient ischemic attack, Minor stroke, Reversible ischemic neurologic deficit, Fatigue, Anxiety, Depression, Post-traumatic stress disorder, Cognitive impairment, Quality of life

## Abstract

**Background:**

Approximately 20,000 people have a transient ischemic attack (TIA) and 23,375 have a minor stroke in England each year. Fatigue, psychological and cognitive impairments are well documented post-stroke. Evidence suggests that TIA and minor stroke patients also experience these impairments; however, they are not routinely offered relevant treatment. This systematic review aims to: (1) establish the prevalence of fatigue, anxiety, depression, post-traumatic stress disorder (PTSD) and cognitive impairment following TIA and minor stroke and to investigate the temporal course of these impairments; (2) explore impact on quality of life (QoL), change in emotions and return to work; (3) identify where further research is required and to potentially inform an intervention study.

**Methods/Design:**

A systematic review of MEDLINE, EMBASE, PsycINFO, CINAHL, Cochrane libraries and grey literature between January 1993 and April 2013 will be undertaken. Two reviewers will conduct screening search results, study selection, data extraction and quality assessment. Studies of adult TIA and minor stroke participants containing any of the outcomes of interest; fatigue, anxiety, depression, PTSD or cognitive impairment will be included. Studies at any time period after TIA/minor stroke, including those with any length of follow-up, will be included to investigate the temporal course of impairments. QoL, change in emotions and return to work will also be documented. The proportion of TIA or minor stroke participants experiencing each outcome will be reported.

If appropriate, a meta-analysis will pool results of individual outcomes. Studies will be grouped and analyzed according to their follow-up timeframe into short-term (< 3 months after TIA/minor stroke), medium-term (3 to 12 months) and long term (> 12 months). Sub-analysis of studies with a suitable control group will be conducted. Exploratory sub-analysis of memory and attention domains of cognitive impairment will be conducted.

**Discussion:**

The current treatment goal for TIA and minor stroke patients is secondary stroke prevention. If these patients do experience fatigue, psychological or cognitive impairments then this treatment alone is unlikely to be sufficient. The results of this comprehensive review will increase understanding of treatment needs for this patient group, identify where further research is required and potentially inform an intervention trial.

## Background

Stroke is caused by an impaired supply of blood to the brain resulting in loss of brain function, and is one of the major causes of mortality and functional disability in the United Kingdom (UK) [1,2]. The severity of strokes differ between patients and can be classified as major stroke, minor stroke and transient ischemic attack (TIA), also known as mini-stroke. The term major stroke broadly refers to strokes that result in long-term or permanent neurological symptoms and may result in disability. Minor stroke is a term that is widely used in research but has not been formally defined, however, it refers to strokes with symptoms lasting longer than 24 hours but where symptoms are mild and non-disabling [3]. TIA is defined by stroke-like symptoms typically lasting less than one hour and no evidence of acute infarction [4]. TIA and minor stroke also create a substantial burden on society and affect a huge proportion of the population. In England, approximately 20,000 people have a TIA and 23,375 people have a minor stroke every year [5].

### Fatigue, cognitive and psychological problems after stroke

In addition to functional disability, sequelae following stroke include fatigue, cognitive impairments and psychological impairments, such as anxiety, depression and post-traumatic stress disorder (PTSD) [6]. These impairments are documented in the literature and have a detrimental impact on people’s lives [7-10]. Fatigue is multidimensional and comprises physical, emotional and cognitive elements [11]. Fatigue has been shown to impact on stroke survivors’ rehabilitation and quality of life (QoL) [12] and is associated with depression [13], inability to return to work [14] and increased fatality post-stroke [15]. The burden of fatigue should not be underestimated, for instance one study found 40% of stroke patients reported fatigue as their worst symptom [16].

Anxiety is universally the most common mental health disorder and is coupled with physical, behavioral and cognitive symptoms [17]. Anxiety and depression frequently occur simultaneously and, in this circumstance, depression is more severe and patients experience higher levels of functional and cognitive impairment [17]. Both major and minor depression have been documented post-stroke and can occur at any time point from the acute stage up to five years post-stroke with an estimated prevalence of 33% [18]. In addition, Ayerbe *et al*. reported that a high proportion of patients with depression post-stroke at one time point remained depressed [19]. Depression is associated with a poor prognosis, decreased QoL and increased mortality [20].

PTSD can develop after exposure to a life-threatening medical event and has been documented post-stroke [21]. Research has shown that occurrence of post-stroke PTSD is independent of physical disability [8]. PTSD has detrimental implications and patients with PTSD have been shown to have an increased risk for a worse physical and mental health prognosis and have greater suicidal intention [8]. It is speculated that a poor health prognosis related to PTSD is associated with both biological mechanisms, such as high blood pressure, and behavioral factors, such as non-adherence to medication [22]. Resultant non-adherence to medication may impact on stroke and TIA survivors as this is essential for secondary prevention of stroke. PTSD also adversely affects people’s QoL and normal functioning [23]. Conversely, life-threatening events can also produce a positive psychological change known as post-traumatic growth. McGrath and Linley [24] reported evidence of sustained positive psychological change after acquired brain injury. However the sample size for this study was small. Furthermore post-traumatic growth following stroke is reported to be inversely correlated with anxiety and depression [25]. This concept is relatively new to stroke research and there is only a small amount of literature available, which to our knowledge has not yet been extended to TIA and minor stroke. Therefore this review will be limited to PTSD.

Cognitive impairment is well documented following stroke and exhibits a wide-range of symptoms including difficulty with memory, reading, writing and number skills, visual impairment and difficulty planning and problem solving. A relationship between cognitive and functional impairment has been reported along with a negative impact on rehabilitative outcomes [26]. Cognitive impairment is associated with depression but the directional relationship is unclear [27]. For the purpose of this review, cognitive impairment will encompass impairments of attention, memory, spatial awareness, perception, apraxia and executive functioning as in accordance with the Royal College of Physicians National Clinical Guidelines for Stroke [28].

### Impairment after TIA and minor stroke

Fatigue, psychological and cognitive impairment have been shown to occur post-stroke in the absence of functional impairment and independent of stroke severity [29]. Although TIA and minor stroke are characterized by short-lasting symptoms, evidence suggests that this cohort experience residual problems. Coutts *et al*. [30] found that 15% of a sample of TIA and minor stroke patients were disabled at 90 days as defined by a modified Rankin Scale score ≥ 2. Another study reported TIA patients to have comparable QoL scores to stroke patients in all domains with the exception of social isolation [31]. However, results of this study may be unrepresentative of the true stroke population as stroke patients in rehabilitation hospitals and care homes were excluded. Significant fatigue has been reported in a community population of TIA and minor stroke patients with a prevalence, at six months, of 29% and 56% respectively [32]. Qualitative research of people’s experiences following TIA or minor stroke revealed that people reported a variety of residual symptoms [33]. These included functional impairments, such as limb weakness and numbness; cognitive impairments, such as memory difficulties; slurred speech; emotional issues, such as feeling depressed, confused and more emotional [34].

Current treatment guidelines relevant to TIA and minor stroke focus on secondary prevention of stroke [5]. However, no consideration is given to psychological or cognitive impacts of TIA or minor stroke and patients are not routinely offered additional rehabilitative support. Untreated fatigue, psychological or cognitive impairment will result in a reduced QoL and affect people’s ability to return to work and social activities.

### Aims

Considering the diversity and complexity of residual impairments anecdotally described by people following TIA and minor stroke, it is important to conduct a comprehensive systematic review of the literature. This is a necessary step to develop future intervention studies that will inform treatment recommendations and guidelines. This systematic review therefore aims to:

•Establish the prevalence of fatigue, anxiety, depression, PTSD and cognitive impairment following a TIA or minor stroke and investigate if this prevalence changes over time.

•Explore the impact of TIA and minor stroke on people’s QoL, change in emotions and return to work.

•Identify where there are gaps in the understanding of residual problems after TIA and minor stroke.

## Methods

Our search strategy, selection of studies, assessment of risk of bias and reporting of results for the review will be conducted in accordance with the Preferred Reporting Items for Systematic Reviews and Meta-analyses (PRISMA) statement [35]. This protocol is not registered with Prospective Registering of Systematic Reviews (PROSPERO) as the scope of the research does not meet their current inclusion criteria.

### Eligibility criteria

The inclusion of studies in the systematic review will be determined by participants, comparators, outcomes and study designs used by the study and the report characteristics.

#### Participants

This review will include TIA and minor stroke participants. Studies will be excluded if they recruited major stroke participants or mixed populations, where it is not possible to extract the data of TIA or minor stroke participants. As stroke is a confounding factor for outcomes of interest, participants must have no previous history of stroke and be stroke free in the follow-up period. Alternatively, the study must have subgroup analysis of stroke free participants. Participants must be adults (over 18 years of age) to exclude childhood stroke. Studies that include participants under 18 years of age will be included if over 90% of the sample are adults.

There is not a standard definition of minor stroke however, as suggested by Fischer *et al*. [3], this patient group should have non-disabling symptoms following stroke but be distinct from TIA patients. TIA patients will be defined by short-lasting stroke symptoms (less than 24 hours) with no evidence of acute infarct. Studies where authors describe their population as TIA, minor stroke, mild stroke, reversible ischemic neurologic deficit or non-disabling stroke will be included.

#### Comparators

Descriptive study designs will be included in this review and, therefore, studies without a comparison group will be included. However, if present, data will be extracted for control groups where participants have no history of stroke, minor stroke or TIA. Participants’ data on outcomes before their TIA or minor stroke will also be used as suitable a comparator.

#### Outcomes

Stroke causes a broad spectrum of impairments; anecdotal evidence from TIA and minor stroke patients emulates these diverse impairments. Given this, exploratory analysis will be conducted to identify the proportion of TIA and minor stroke participants with the following; fatigue, anxiety, depression, PTSD or cognitive impairment. These principle outcomes will be defined by scores above the predefined cut off limit for validated assessments. Studies must either report the frequencies for outcomes or data whereby frequencies can be calculated. There will be no restrictions on the duration of participant follow-up or time since TIA or minor stroke to develop understanding about the temporal course of the outcomes. Information on QoL, change in emotions and return to work or performance at work will also be documented. Studies will be included that report any of the above outcomes.

#### Study design

Initial scoping searches ascertained that a limited amount of relevant studies have been conducted that include a comparator group. Therefore, all study designs will be included with the exception of single case studies and reviews. Intervention studies may be included if the non-intervention arm consists of TIA or minor stroke patients receiving usual care. Only data from this control arm will be analyzed as this review is not investigating interventions.

#### Report characteristics

Only human studies will be included. Full papers, conference abstracts and theses will be included. To avoid language bias, non-English papers will be included. For pragmatic and quality of reporting reasons, the review will limit the search to 20 years (1993 to 2013).

### Information sources

Electronic searches of the following databases will be conducted; MEDLINE, EMBASE, PsycINFO, Cumulative Index to Nursing and Allied Health Literature (CINAHL), Database of Abstracts of Reviews of Effects (DARE), the Cochrane Central Register of Controlled Trials (CENTRAL) and Cochrane Database of Systematic Reviews (CDSR). Databases will be searched from January 1993 to April 2013.

Ongoing studies will be identified by searching clinicaltrials.gov, Stroke Trials Registry (http://www.strokecenter.org/trials/) and conference abstracts including the American Heart Association International Stroke conference, European Stroke conference and UK Stroke Forum. Conference Proceedings Citation Index (CPCI) will also be searched for conference abstracts. These sources will be searched from 2010 to 2013 as it will be assumed studies presented before these dates will have been completed and published.

Grey literature will be explored including PubliCAT and ScienceDaily.com. The first four pages of Google Scholar results will be searched; this limit was established from scoping searches. Dissertations and theses will be identified from the databases ProQuest Dissertation Thesis Database and thesis.com. References from included studies will be scanned and tracked through the cited reference search in Science Citation Index (SCI).

### Search

A comprehensive search strategy has been developed that focuses on the following elements; TIA, minor stroke, fatigue, anxiety, depression, PTSD and cognitive impairment. Search terms have been established by scoping searches. The MEDLINE search strategy is detailed in Additional file 1. This search will be adapted for the other databases.

### Study selection

The titles and abstracts of the search results will be screened and full text will be obtained for relevant studies. Two authors (GT and BF) will complete this process independently and any difference in opinion will be resolved by a third reviewer (TM). Full text articles will be reviewed to determine if studies included through screening meet the inclusion criteria. An inclusion checklist has been developed, based on the eligibility criteria, and piloted (Table 1).

**Table 1:** The inclusion checklist for screened references

Study design	Cross sectional
	Cohort
	Case control
	Case series
	Other (please specify)
	Study objectives relevant to topic
Report characteristics	Full article
	Conference abstract
	Thesis
	Other (please specify)
	Publication date 1993 to 2003
Participants	TIA
	Minor stroke
	Study sample are adults
	Participants have no previous history of stroke/subgroup analysis of those with no history of stroke
	Participants stoke free during follow-up/subgroup analysis of those stroke free
Comparator (Do NOT exclude if no comparator)	Comparator group present? (If no, go to outcomes)
	No previous history of stroke, minor stroke or TIA
Outcomes	Measure for anxiety
	Measure for depression
	Measure for fatigue
	Measure for PTSD
	Measure for cognition
	Quality of life reported
	Change in emotions reported
	Return to work reported
	Frequencies reported/can be calculated

### Data collection process

Data extraction will be performed in duplicate by two independent reviewers (GT and BF) for all of the eligible papers identified through the screening process. A data extraction form has been developed which focuses on population, comparator, outcomes and study design. This form has been informed by the STROBE (STrengthening the Reporting of OBservational studies in Epidemiology) statement [36]. The data extraction form was piloted independently by two researchers (GT and MF) on known papers. A Microsoft Excel document will be used to manage the data extraction.

### Quality assessment

Quality assessment of included studies will be performed by two independent reviewers (GT and BF). A quality assessment form has been devised which focuses on sampling, measurement of outcomes, attrition and analysis (Table 2). This form has been piloted independently by two researchers on known papers (GT and MF). In accordance with the Cochrane Collaboration recommendations, an overall score will not be generated but a risk of bias judgment of ‘yes’, ‘no’ or ‘unclear’ will be given for individual domains [37]. The studies level of quality will be presented in a table and narrative summary. The impact of the studies quality on results will be evaluated in the discussion. If appropriate, a sensitivity analysis will be conducted excluding poor quality studies.

**Table 2:** The quality assessment criteria for included studies

		**Judgment (yes/no/unclear)**
		**Support for judgment**
Sampling	Was the study design appropriate to answer the research question?	
	Was the sampling method appropriate?	
	Did the study report how many people were approached and how many agreed to take part?	
	Do those that participate have similar characteristics to those that refused (for example, age, gender, comorbidities, how they were approached)?	
	Is the sample size adequate?	
	Did the study describe how the sample size was determined?	
	Was a suitable definition of TIA/minor stroke used?	
	If applicable, was the control group comparable to cases (consider suitability, recruitment and baseline characteristics)?	
	Did the study demonstrate if the outcomes were present before the TIA/minor stroke (for example, history of depression)?	
Measurement	Was a suitable measurement for outcome used?	
	Has the outcome measure been validated for the population?	
	Was the outcome measure cut-off score predefined?	
	Was the outcome measure administration suitable (for example, self reported, investigator interview)?	
	Were potential confounding variables measured?	
Attrition	Were numbers of dropouts/withdrawals documented at each time point?	
	Were reasons given for dropouts/withdrawals?	
Analysis	Were all outcomes reported?	
	Were confounding variables adjusted for?	

### Synthesis and analysis of results

If the outcomes demonstrate homogeneity, the results for individual outcomes will be pooled quantitatively using meta-analysis. The temporal course of fatigue, psychological and cognitive impairment post-stroke has not been well characterized. However, prevalence of post-stroke depression has been shown to have immediate onset, peak at three to six months but also remain high years after stroke [38]. To explore the time course of these impairments following TIA and minor stroke, studies will be grouped into short-term (less than three months after TIA or minor stroke), medium-term (three to twelve months) and long-term (over twelve months). If appropriate, frequencies for each outcome will be combined to create pooled estimates for short, medium and long term timeframes.

A sub-analysis of studies with a suitable control group will also be conducted to determine whether outcome proportions are higher in TIA and minor stroke patients compared to healthy controls. Cognitive impairment covers a spectrum of different domains. Anecdotal evidence suggests that TIA and minor stroke patients experience residual memory and attention complaints. Therefore, exploratory analysis of specifically memory and attention domains of cognitive impairment will be conducted. To investigate the natural history of fatigue, psychological and cognitive impairment after TIA and minor stroke, exploratory analysis of the outcomes for new cases compared to persistent cases will be conducted for studies with more than one time point.

If studies are methodologically heterogeneous, a narrative synthesis of results will be more appropriate. In accordance with the Center for Reviews and Dissemination (CRD), included studies will be summarized in a table detailing study type, sample size, participant characteristics, outcomes and outcome measures [39]. The Economic and Social Research Council (ESRC) guidance report will be used as a framework for a narrative synthesis [40].

## Discussion

Currently the treatment goal for TIA and minor stroke patients is the prevention of subsequent stroke [5]. These patients are not acknowledged to have residual problems which require management. This systematic review will collate literature to establish whether evidence suggests people experience fatigue, anxiety, depression, PTSD or cognitive problems following a TIA or minor stroke. The temporal nature of the prevalence of these impairments after the event will also be investigated. In addition, impact of TIA and minor stroke on QoL, change in emotions and return to work will be explored. This systematic review will identify if there is a lack of literature for any of the outcomes and review the quality of the available evidence.

### Implications of results

If it is found that TIA or minor stroke patients have an increased prevalence of fatigue, psychological or cognitive problems, then the current management, without addressing them, is unlikely to be adequate. Dissemination of results will increase the awareness of the treatment needs of TIA and minor stroke survivors. This information will be particularly valuable in the primary care setting where it is likely that this patient group will present with residual complaints.

The comprehensive and systematic search and review of the literature will identify and inform where further research is required. For instance, if a large number of descriptive studies are available for the review, then the findings will inform a subsequent analytical, hypothesis testing, study. Alternatively, the findings from the review might indicate that exploration research has already been conducted and will therefore inform the design of an acceptability and feasibility study. This subsequent study will investigate the effects of an intervention to manage and treat fatigue, psychological and cognitive problems following TIA and minor stroke.

## Abbreviations

CDSR: Cochrane Database of Systematic Reviews; CENTRAL: Cochrane Central Register of Controlled Trials; CINAHL: Cumulative Index to Nursing and Allied Health Literature; CPCI: Conference Proceedings Citation Index; CRD: Center for Reviews and Dissemination; DARE: Database of Abstracts of Reviews of Effects; ESRC: Economic and Social Research Council; PRISMA: Preferred Reporting Items for Systematic Reviews and Meta-analyses; PROSPERO: Prospective Registering of Systematic Reviews; PTSD: post-traumatic stress disorder; QoL: quality of life; SCI: Science Citation Index; STROBE: STrengthening the Reporting of OBservational studies in epidemiology; TIA: transient ischemic attack; UK: United Kingdom.

## Competing interests

The authors declared that they have no competing interests.

## Authors’ contributions

GT drafted the manuscript. TM, MC, MF and CS provided feedback on the manuscript. GT, TM, MC, MF and CS were involved in the design of the review. GT conducted scoping searches, GT and MF piloted the inclusion/exclusion, quality assessment and data extraction forms. GT will be first reviewer and BF will be the second reviewer for the systematic review. All authors read and approved the final manuscript.

## Supplementary Material

Additional file 1 **Search strategy for MEDLINE (via Ovid) January 1993 to April 2013.** Full electronic search strategy for MEDLINE. Click here for file
