# Supplementary material for: A systematic review investigating fatigue, psychological and cognitive impairment following TIA and minor stroke: protocol paper
Source: Syst Rev. 2013 Sep 8;2:72. doi: 10.1186/2046-4053-2-72 (PMC3846122; doi:10.1186/2046-4053-2-72)
Supplement: Additional file 1 — Search strategy for MEDLINE (via Ovid) January 1993 to April 2013. Full electronic search strategy for MEDLINE. [file 2046-4053-2-72-S1.pdf]

Additional file 1- Search strategy for MEDLINE (via Ovid) 1993 to April 2013

1. transient isch?emic attack$.mp. or exp Ischemic Attack, Transient/
2. TIA.mp.
3. TIAs.mp. [mp=title, abstract, original title, name of substance word, subject heading word, keyword heading word, protocol supplementary concept, rare disease supplementary concept, unique identifier]
4. (transient adj (brain isch?emia$ or cerebral isch?emia$ or CVA$ or cerebral vasc$ or cerebro vasc$)).mp. [mp=title, abstract, original title, name of substance word, subject heading word, keyword heading word, protocol supplementary concept, rare disease supplementary concept, unique identifier]
5. mini stroke.mp.
6. minor stroke$.mp.
7. mild stroke$.mp.
8. NDS.mp.
9. non$disabling stroke$.mp.
10. (minor adj (cerebrovasc$ accident$ or cerebrovasc$ stroke$ or CVA$ or brain isch?emia$ or cerebral stroke$)).mp. [mp=title, abstract, original title, name of substance word, subject heading word, keyword heading word, protocol supplementary concept, rare disease supplementary concept, unique identifier]
11. Non$severe stroke$.mp. [mp=title, abstract, original title, name of substance word, subject heading word, keyword heading word, protocol supplementary concept, rare disease supplementary concept, unique identifier]
12. RIND or reversible isch?emic neurologic$ deficit$
13. 1 or 2 or 3 or 4 or 5 or 6 or 7 or 8 or 9 or 10 or 11 or 12
14. "quality of life".mp. or exp "Quality of Life"/
15. exp Anxiety/ or exp Anti-Anxiety Agents/ or exp Anxiety Disorders/ or anxiety.mp.
16. exp Stress Disorders, Post-Traumatic/ or stress disorder$.mp.
17. (anxiety disorder$ or agoraphobia$ or obsessive$compulsive disorder$ or panic disorder$ or phobic disorder$ or distress$ or panic$).mp. [mp=title, abstract, original title, name of substance word, subject heading word, keyword heading word, protocol supplementary concept, rare disease supplementary concept, unique identifier]
18. (feel$ adj3 (apprehens$ or dread or disaster$ or fear$ or worry or worried or terror)).mp. [mp=title, abstract, original title, name of substance word, subject heading word, keyword heading word, protocol supplementary concept, rare disease supplementary concept, unique identifier]
19. exp Depression/ or depress$.mp.
20. exp Antidepressive Agents/ or antidepress$.mp. or exp Depressive Disorder/
21. mood disorder$.mp. or exp Mood Disorders/
22. posttraumatic stress disorder$.mp.
23. post traumatic stress disorder$.mp.
24. PTSD.mp.
25. (flashback$ or avoidance$ or avoid$ or re$experience).mp. [mp=title, abstract, original title, name of substance word, subject heading word, keyword heading word, protocol supplementary concept, rare disease supplementary concept, unique identifier]

26. exp Cognition Disorders/ or cognit$ disorder$.mp. or exp Cognition/
27. exp Attention/ or cognit$ impair$.mp.
28. (cognition or orientation or attention or perception or mental processing or problem solving or memory).mp. [mp=title, abstract, original title, name of substance word, subject heading word, keyword heading word, protocol supplementary concept, rare disease supplementary concept, unique identifier]
29. exp Fatigue/ or exp Fatigue Syndrome, Chronic/ or fatigue.mp.
30. CFS.mp. [mp=title, abstract, original title, name of substance word, subject heading word, keyword heading word, protocol supplementary concept, rare disease supplementary concept, unique identifier]
31. (fatigue adj (chronic or syndrome$)).mp. [mp=title, abstract, original title, name of substance word, subject heading word, keyword heading word, protocol supplementary concept, rare disease supplementary concept, unique identifier]
32. 14 or 15 or 16 or 17 or 18 or 19 or 20 or 21 or 22 or 23 or 24 or 25 or 26 or 27 or 28 or 29 or 30 or 31
33. 13 and 32
34. limit 33 to (humans and yr="1993 -Current")
